# To What Extent Are Informal Healthcare Providers in Slums Linked to the Formal Health System in Providing Services in Sub-Sahara Africa? A 12-Year Scoping Review

**DOI:** 10.1007/s11524-024-00885-5

**Published:** 2024-06-14

**Authors:** Aloysius Odii, Ifeyinwa Arize, Prince Agwu, Chinyere Mbachu, Obinna Onwujekwe

**Affiliations:** 1 Health Policy Research Group, Department of Pharmacology, College of Medicine, University of Nigeria, Enugu Campus, Enugu, Nigeria; 2 Sociology/Anthropology Department, Faculty of the Social Sciences, University of Nigeria, Nsukka, Nigeria; 3 Health Administration and Management Department, Faculty of Health Sciences & Technology, College of Medicine, University of Nigeria Nsukka, Enugu Campus, Enugu, Nigeria; 4 Social Work Department, Faculty of the Social Sciences, University of Nigeria, Nsukka, Nigeria; 5 Department of Community Medicine, Institute of Public Health, College of Medicine, University of Nigeria Nsukka, Enugu Campus, Enugu, Nigeria

**Keywords:** Informal health providers, Formal health providers, Slums, Health systems

## Abstract

The contributions of informal providers to the urban health system and their linkage to the formal health system require more evidence. This paper highlights the collaborations that exist between informal providers and the formal health system and examines how these collaborations have contributed to strengthening urban health systems in sub-Sahara Africa. The study is based on a scoping review of literature that was published from 2011 to 2023 with a focus on slums in sub-Sahara Africa. Electronic search for articles was performed in Google, Google Scholar, PubMed, African Journal Online (AJOL), Directory of Open Access Journals (DOAJ), ScienceDirect, Web of Science, Hinari, ResearchGate, and yippy.com. Data extraction was done using the WHO health systems building blocks. The review identified 26 publications that referred to collaborations between informal providers and formal health systems in healthcare delivery. The collaboration is manifested through formal health providers registering and standardizing the practice of informal health providers. They also participate in training informal providers and providing free medical commodities for them. Additionally, there were numerous instances of client referrals, either from informal to formal providers or from formal to informal providers. However, the review also indicates that these collaborations are unformalized, unsystematic, and largely undocumented. This undermines the potential contributions of informal providers to the urban health system.

## Introduction

Globally, the 4.4 billion people who live in cities account for about 56% of the world’s population [1]. It is projected that urban population will increase to 68% by 2050, and approximately 90% of this increase will take place in Asia and Africa [2]. The increase in urban population creates many challenges, notably the sprawling of slums due to shortages of affordable housing [3]. Slums (interchangeably used with informal settlements) are characterized by unplanned and dilapidated housing, with deprivation and constraints on healthcare services. In sub-Sahara Africa, most (55%) urban dwellers now live in slums and are deprived of access to basic amenities and quality healthcare services [4].

As cities in sub-Sahara Africa continue to grow, one of the main challenges in the future will be how to provide access to healthcare services, which nowadays represents a key component of development [3]. Sustainable Development Goal 10 calls for the reduction of inequality between and within countries. However, inequality in access to health continues to increase even in urban areas and contributes to a high rate of maternal and child mortality among vulnerable groups [5]. Cities thrive when everyone is productive, so when people are excluded from access to healthcare, they would not be able to produce at their maximum.

There is the neglect of urban health-focused policies and this contributes to the widening health disparity in urban areas [6, 7]. Health outcomes for urban slum dwellers and non-slum dwellers differ; slum dwellers are more likely to experience undernutrition, higher child mortality rate, and higher transmission rate of infectious diseases [8]. Additionally, urban slum dwellers experience poorer access to healthcare services and relatively lower presence of formal health providers. Hence, they bear the brunt of inequalities in urbanization [9]. Studies have shown that when formal healthcare is lacking in urban slums, residents turn to informal healthcare providers [10, 11].

Strengthening healthcare delivery in urban areas, especially in urban slums in sub-Sahara Africa, involves holistic actions across the health system building blocks [12, 13]. Furthermore, strategies to improve responsiveness of urban health systems have been identified to include adopting a multi-sectoral approach, citizen’s participation, recognition of diverse service providers, and use of evidence [14]. This implies the recognition that patients are not passive receivers of health services, but their experiences, views, and decisions have the capacity to improve overall health outcomes [15]. Thus, strengthening health system may involve the recognition of healthcare services, such as those provided by informal providers, which are available to serve population needs.

Informal healthcare providers include a diverse group of practitioners who provide services for which they have no formal medical training or are not licensed to provide [16]. They include patent medicine vendors (PMVs), traditional birth attendants (TBAs), herbalists, traditional healers, and informal allopathic providers (IAPs). They have enjoyed high patronage in urban slums because they provide a wide range of promotive, preventive, and curative services that are convenient, affordable, and appeal to the social and cultural milieu of the people [17]. However, they do not enjoy legitimacy like the formal health sector because their practitioners are not formally trained to provide the services they provide [18].

Although estimates of the size of the informal healthcare sector vary, it is thought that informal providers account for a sizeable fraction of all healthcare providers in sub-Sahara Africa and deliver a sizeable portion of care in the region [17]. The role of informal health service providers has been recognized in numerous legislative and programmatic activities. Considering their population, spread, and easy access, informal providers could be the key in the effort to meet some of the health-related sustainable development goals, particularly in underserved urban slums. However, their services and health data have yet to be incorporated into the formal healthcare system [11, 19]. Serious efforts are needed to advance a clearer definition of the informal provider’s roles and responsibilities in providing services, disseminating health-related information, and even participating in the co-regulation of pharmaceutical and healthcare markets.

The objective of this review is to synthesize evidence in urban slum literature on existing relationships and collaborations between informal providers and the formal health system to document how this contributes to health system strengthening. This was achieved by employing the WHO health systems building framework described in Table 1 [20]. This will inform ways for policymakers to properly link informal providers to formal health systems and reduce health disparities within cities.

**Table 1:** Description of WHO health systems building framework

Leadership and governance	Service delivery	Health workforce	Medical products, vaccines and technology	Health financing	Health information
We included text that provides information on how the collaboration between IHF and FHP affected effective oversight, accountability, and regulation of practice	We included text that provides information about how collaborations between IHP and FHP influenced equitable access to care	We included texts that describe how collaborations between IHP and FHP influenced the availability of the health workforce as well as their competence, responsiveness and productivity	We included text which looked how collaborations between IHP and FHP influenced equitable access to essential medical products, vaccines, and technologies	We included text that described how collaborations between IHP and FHP influenced funding for health	We included text which described how the collaborations between IHP and FHP influenced the production, analysis, and dissemination of health information

## Methodology

A scoping literature review was undertaken to examine the extent to which formal and informal providers collaborate in the provision of care. Our literature search was based on the Population, Concept, and Context (PCC) [21]. The “Population” included informal providers and formal providers; the “Concept” was collaborations; and the “Context” was urban areas or urban slums in SSA.

We operationalized collaborations as relationships that exist between formal and informal health providers aimed at providing quality health services to the population [11]. Such relationships may be direct or indirect and initiated by any of the providers, patients, or other external bodies (i.e., NGOs). The search was conducted in Google, Google Scholar, PubMed, African Journal Online (AJOL), Directory of Open Access Journals (DOAJ), ScienceDirect, Web of Science, Hinari, ResearchGate, and yippy.com. We also expanded our search to the websites of relevant ministries, departments, agencies, and organizations.

## Inclusion Criteria

Documents were included if they met the following criteria: [1] published journal articles, policy documents from Nigeria alone (including strategic plans), media reports, and grey literature; [2] besides classical/historical literature, all documents were from 2011 to 2013 written in English language; [4] the literature covered sub-Sahara Africa with particular emphasis on urban areas and slums in Nigeria; [5] refers to collaborations or linkages between informal providers and formal health system; and [6] refers to any of the WHO health system building blocks.

The choice of the timeframe was to allow for a comprehensive examination of the recent literature in the field, as there is still limited evidence on collaborations between the informal providers and the formal health systems. This longer time frame allowed the researchers to gather useful evidence, showing that research on this subject has gained momentum in recent years. Additionally, the focus on sub-Sahara Africa is because of the region’s unique healthcare landscape characterized by poor healthcare delivery and a high prevalence of informal healthcare providers [17]. The emphasis on Nigeria is because, besides being the most populous country in Africa, Nigeria presents a significant case study for understanding the dynamics between informal and formal healthcare systems within the context of sub-Sahara Africa [22].

## Data Extraction and Charting

The methodological guidance for the conduct of scoping reviews guided the extraction and charting of relevant data [21]. This was done using Microsoft Excel by the following authors: AO, IA, PA, and CM. The information elicited from the source and placed in an Excel spreadsheet included: author(s), year of publication, country of study, aims of the study, study population, research design, extent of collaborations between informal providers, and the formal health system using WHO building blocks. The data were extracted after a thorough assessment.

The literature search and mining were guided by the following stages:

- Stage 1: Generation of search terms and electronic search in various search engines and databases.
- Stage 2: Removal of duplicates of articles.
- Stage 3: Screening of articles for eligibility.
- Stage 4: Extraction of relevant contents into the data mining template.
- Stage 5: Summarization of findings from each article/source.
- Stage 6: Merging of findings from individual reviewers.

## Data Analysis and Synthesis Methods

Narrative synthesis of findings was done for each health system building block in which informal providers collaborate with the formal health system. Key concepts emerging from the included studies were grouped and categorized to illustrate a broader picture. This method entails synthesizing various concepts to systematically address a central theme, effectively tackling the research questions [23]. In this case, the themes evolved from the WHO health system building blocks which include leadership/governance, health financing, service delivery, health workforce, medical products, vaccines, and technologies and information. The information in each cell was extracted based on the building blocks they are addressing.

## Findings

**Fig. 1:**
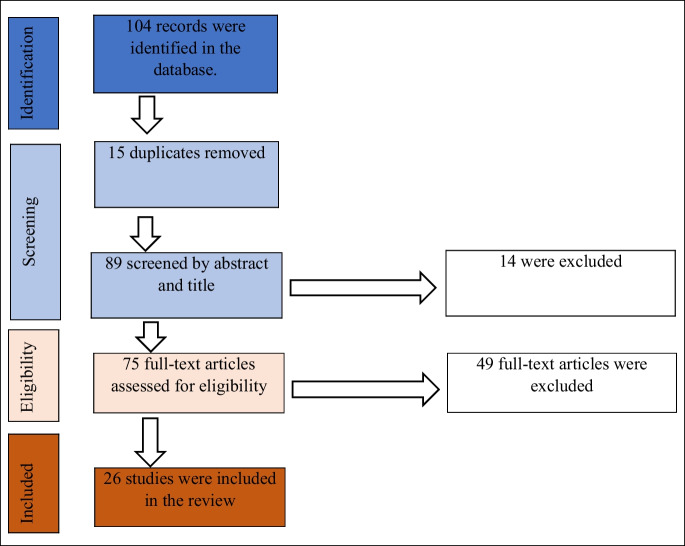
Flow chart showing article extraction and inclusion method

We retrieved 104 academic and grey literature for screening. Fifteen [15] duplicates were removed, and 89 articles were screened using the eligibility criteria. Seventy-five (75) articles were assessed for eligibility, and only 26 articles were eligible for the review. A detailed representation of the review process is shown in Fig. 1. The findings from the review are presented according to the WHO building blocks.

## Leadership and Governance

Governance and leadership were poorly represented in the literature included in the review. There are few examples of coordination and regulation by associations and agencies of governments. For example, in Nigeria, informal providers like Patent and Proprietary Medicine Vendors (PPMVs) and retail pharmacies obtain their licenses from the Pharmacists Council of Nigeria (PCN) [24]. Meanwhile, supervision and monitoring of PPMVs are carried out by PCN and the State Ministry of Health (SMH). These have built trust and have encouraged the reporting of itinerant drug sellers and the reporting of unregistered, unlicensed, and non-compliant PPMVs to the regulatory bodies [25]. In Uganda, however, informal providers are appreciated locally, but the ministry of health are yet to formalize their recognition [26].

Patent and Proprietary Medicine Vendors (PPMVs) through their various activities also provide governing frameworks for their members. They organize workshops where they invite qualified pharmacists and health workers to engage their members. Moreover, they register and monitor them as well as promote and defend their interests if the need arises. Those who do not adhere to their rules such as sales of prescription medications could lose their membership [16].

It was reported that collaboration between informal and formal health systems can improve better regulation and monitoring of practices and products [11]. Monitoring and supervision can be carried out by the Ministry of Health or any authorized government representatives. However, some informal providers, particularly PPMVs fear that linking informal providers to the formal health system, could open doors for increased taxation, loss of clientele, disparagement by the formal health system, and limitation of practice.

In Nigeria, local health committees were shown to conflict with the informal healthcare providers regarding where patients should seek care [27]. Local health committees successfully mobilized community members to prefer utilizing primary healthcare facilities over informal healthcare providers. The reasons were founded on the competence of service delivery and the incentivization of the local health committees by the formal health institution.

## Service Delivery

Documents included in the review showcase how informal and formal providers collaborate in the provision of care. Health seekers obtain care from informal and formal providers, and they may do so without full disclosure to both parties involved. In one study, the non-disclosure rate ranged from 55.8% to 100%, with an average of 83% [28]. Further, reasons for non-disclosure include fear of receiving improper care and a negative attitude towards informal providers. In a study in Nigeria, it was found that about 52.9% of mothers combine seeking care in health facilities with patronizing providers who utilize unorthodox practices in the provision of care [29].

A study in Uganda and Sudan also showed that patients with mental health issues and psychosis sought the services of Traditional Complementary and Alternative Medicine (TCAM) prior to accessing care from an approved health institution [28]. The attitude of health workers in a formal health system can also make patients switch from accessing care in formal health institutions to informal providers [27, 30].

But in other examples, it is the cost of care as well as other challenges such as stress, delay, and absenteeism of health workers that motivate health seekers to switch to informal providers [27, 30]. The price of care in public hospitals can sometimes double that of informal providers in urban slums [31]. When that occurs, some health seekers prefer to access care from informal providers. TBAs are an example of informal providers sought after by health seekers. This is because they have been shown to charge less as well; they offer health seekers financial accommodation and room for instalment payments. The flexibility of the informal health providers regarding payment opens the door for increased patronage by health seekers in urban slums.

Informal providers reportedly offer a range of care and in fact; they act as healthcare providers [32]. They counsel patients prescribe drugs and conduct delivery and diagnostic care. Then, [33] reported that TBAs offer delivery services, nutritional counselling, family planning, and contraceptives, as well as other supports to their patients. Similarly, PPMVs (or chemists) also handle ailments and dispense drugs as the first-line source of healthcare [19].

Informal providers also participate in health promotion activities. TBAs are engaged in HIV case identification and referrals, and PPMVs engage in malaria diagnosis and treatment [11].

Formal health providers also negotiate with informal providers to refer some patients to their facilities. Informal providers are expected to be altruistic since they are highly patronized by urban slum dwellers [34]. However, as was shown in the Ebola virus in Monrovian slums, mistrust among providers contributed to misinformation and treatment delays of informal providers [35].

In one study, PPMV respondents indicated that the identification of cases they considered beyond their capacity was also the primary criterion they used to decide whether to refer a customer to a more highly trained healthcare provider [19]. Most providers reported that after dispensing medicines, they would refer any customer who did not improve within two or three days.

## Health Workforce

The documents included in the reviews underscore critical shortages of formal health providers, especially in urban slums [16, 24]. Also, it was reported that informal providers have been filling this gap by treating common health problems [36] and providing health services for underserved population [11].

In response to this, regulatory agencies like National Agency for Food and Drug Administration and Control (NAFDAC) and National drug Law Enforcement Agency (NDLEA) were said to provide seminars on the sale of appropriate drugs, consultative meetings with the executives of PPMV, and state governments conduct training through NGOs [25].

Although informal providers are available and might even help resolve the challenge of scarcity of formal providers in urban slums, their competence is often called into question. The review recorded minimal actions by the government towards improving the competence, responsiveness, and productivity of informal providers. Therefore, informal providers had to rely on several sources, including the media and pharmaceutical representatives for health-related expert knowledge [16]. PPMV associations are known to organize training for their members and oversee adherence to certain treatment standards to improve their competence and recognition by the formal health system. The challenge with increasing the competence and productivity of informal providers is that PPMV licensure does not require formal training in medicine or pharmacy; many PPMVs complete an apprenticeship with a more senior PPMV before opening their own shop [24]. Moreover, they rarely follow the advice which could lead to reduced income or clients [16].

There are other instances where informal health providers make the move to be recognized by the formal health system. In South Africa, through the Valley Trust in Kwazulu-Natal, informal providers volunteered to be part of the community health workforce and were trained for three years to qualify as Community Health Workers (CHWs) [37]. Since then, they have continued to provide health services in areas lacking public health facilities and are in communication with the formal health system. A similar pattern of relationship is observed in other low-income countries where workshops are organized for traditional healers on healthcare and the use of antibiotics, and consumables for the prevention of sexually transmitted diseases (STDs) and other diseases are given to them for distribution to their service users [38].

Additionally, there are cases where providers are linked to both formal and informal healthcare. Sieverding and Beyeler [19] reported that community health workers employed in government establishments moonlight as PMVs, making it difficult to sometimes differentiate the formal from the informal providers. Most drug vendors in the northern states of Nigeria have undergone formal medical training which empowers them to deliver high-quality health services and thereby complement the existing healthcare infrastructure [39].

Some PPMVs have formal health training, and some are even simultaneously working in health centers and hospitals. These members tend to be more knowledgeable of healthcare practices and more conversant with regulatory requirements [36]. PPMV owners who have worked or are still working in health facilities thought that they are qualified to prescribe drugs following years of practice or working with healthcare professionals [40]. But such services were examined and found to have fallen short of expectations in prescriptions, referral, and management of side effects that may arise during treatments. On that note, Nelissen et al. [41] argue that informal providers in Nigeria will perform better if they are properly integrated into the formal health system.

## Medical Products, Vaccines, and Technologies

Documents included in the review underscore lack of access to essential medical products, vaccines, and technologies by informal providers. But it also showed how the collaborations are resolving this challenge. One study reported that informal providers are trained and given supplies like antibiotics, condoms, and birth kits to improve service delivery [38]. Collaborating this, a study in Uganda shows that drug shop owners, along with healthcare providers, are provided with antibiotics like metronidazole to treat diarrhea and stomach pain [42]. It was also reported that informal providers are somewhat incentivized through gifts, commissions, small medical equipment, and medicine samples by the formal providers to refer patients to them [34].

Some informal providers like TBAs who lack medicines and other supplies reportedly encourage nursing mothers to take their children to public hospitals for examination or to receive routine immunization [33]. Such referrals arise when they perceive that they cannot handle a case or do not have the needed equipment or materials to tend the patients’ needs. Also, referrals are made from PPMV to public health facilities for treatment that demands crucial medical products and technologies. Notably, referrals are discouraged by stockout of medical products in formal health facilities [43].

Health facilities follow laid down procedures to procure drugs and other technologies, but this can lead to the unavailability of medicines and technologies to attend to patients. Sieverding and Beyeler [19] reported that when drugs are lacking in health facilities, some health workers proceed to PPMV to either fill their stock or to purchase drugs for patients with special needs. Referrals from public health facilities to PPMVs for drug purchase is a common practice but health workers may not always have direct contact with PPMVs. Health seekers would also at times be referred to PPMV shops for drug purchases if the necessary medications were unavailable at the health facility.

There were also instances where ailments were attributed to the supernatural and available medicines and technologies were seen as insufficient to properly diagnose and treat such ailments. On such occasions, orthodox doctors reportedly refer medical cases beyond their comprehension to the traditional practitioners [44].

## Health Financing

While there is no evidence of how collaborations between informal and formal providers benefit patients to access services without suffering financial hardship, there is evidence of informal providers being incentivized (financially or otherwise) to refer clients to formal providers. Sulayman and Adaji [45] reported that when TBAs refer cases to health facilities, they are offered training in alternative jobs including soap manufacturing, catering, and tie-dying, as well as microfinance loans to help them start small enterprises. Other programs adopted the technique of reimbursing TBAs for transportation and other costs previously incurred in the patient's care prior to referral [46].

## Discussion

The review evidences the fact that informal providers and formal providers collaborate to provide healthcare services in urban slums. Examples exist of roles played by informal providers even as they fill the gap for formal health providers as well as collaborate with them to prevent illness, treat disease and infection, and promote the health of urban slum dwellers. When public facilities are unavailable as a result of its distance from urban slum dwellers due to uneven distributions, informal providers enjoy high patronage because they are easily accessible, affordable, culturally inclined, and are highly regarded within the community they are domiciled [33].

However, they are limited in knowledge about appropriate medical practices and treatment guidelines for most illnesses, which is why institutionalizing their relationship with the formal health system would serve to improve healthcare outcomes in urban slums [11]. The literature included shows that the relationship between informal and formal health providers has been unpredictable and largely unregulated.

All the WHO building blocks, with the exception of health information, mirrored ways through which collaborations of formal and informal providers contribute to health system strengthening. Using WHO building blocks reveals the gains of these collaborations and areas to be prioritized. The government must now oversee and guide the direction of this collaboration. Presently, PPMVs obtaining licenses from PCN and conducting training for their members are not sufficient [24] as well as receiving seminars and trainings through NGOs [25]. It is clear from the review that linking the informal providers to the formal health system will require a clearly written policy on how these collaborations will work as well as clearly defined roles of local authorities like community stakeholders who are instrumental to health governance at the local level [27].

Referrals from informal providers to formal providers remain an important and reoccurring aspect of the collaborations between informal and formal providers [11, 27, 34]. However, in its current form, it is not documented, regulated, or follow any laid down procedure. Patients who have previously received care from informal providers may opt to seek services from formal health professionals without being referred (James et al., 2018). Patients may also opt to receive care from informal providers due to cost and other health system challenges like delay and absenteeism of health workers [27, 30, 31, 33]. Informal providers may also refer when incentivized with financial and non-financial rewards [45]. For referrals to be effective, patients must be referred to the right place, at the right time and with the right information.

It is believed that institutionalizing the relationships between informal and formal providers may strengthen the health system of resource-constrained countries. However, lack of trust in the services of informal providers may hinder the chances of institutionalizing a linkage of informal health providers to the formal health system [31]. Also, the fear that linkages may threaten the existence of informal providers is acknowledged [11]. Moreover, there is the need to weigh the cost and benefits as well as their impact on patients’ health expenditures. This needs to be explored as well. There is also the need for more evidence on how these collaborations improve health outcomes, especially for urban slum dwellers. Only one study evidenced improved service coverage as a result of linking informal providers to the formal health system [37].

## Strength

This scoping review adds to a body of literature on the extent of collaborations between informal and formal healthcare providers to highlight future areas of focus for health system strengthening. Although the WHO framework of health system building block was not originally developed for this, it is reported by different authors as an important framework for research on health systems, and it has been utilized in several reviews (47, 48). This study further shows that the framework is important for organizing the collaborations between formal and informal health providers in sub-Sahara Africa.

This review included diverse evidence from different study designs, including qualitative, quantitative and mixed-method studies. It also included multiple perspectives from healthcare providers, policymakers and community actors, leading to rich and diverse evidence.

The findings of the review may have policy implications for policymakers in sub-Sahara Africa. The review informs the need to strengthen the collaborations between informal healthcare providers and formal health providers, as a strategy for improving access to quality healthcare services, particularly for vulnerable populations who reside in slums. Although the literature highlights key players likely to drive the collaborations between the informal and formal health providers, their activities were mainly described as separate actions. Thus, it is necessary to think through a partnership among different bodies and agencies, seeing that challenges to collaboration between informal health providers and the formal health system are multifactorial.

## Limitations

There are some limitations in our review. First, our searches covered a large number of databases over an extensive period. However, despite the extensive search, some studies may have been missed. Second, some of the studies in the review included limited sample size, eternal validity and generalizability which may be a concern. Hence, the extrapolation and implications of our findings may be limited. Third, the wide variations in study designs and the types of providers included in the review; the heterogeneity of the sector was not fully explored. Finally, the review did not pay closer attention to exploring the interrelationship between the building blocks but tried to report them as separate elements.

## Conclusion

The presence of collaborative efforts between formal health providers and their informal counterparts is evident across the WHO building blocks. In light of the scarcity of public health facilities within urban slums, coupled with a substantial number of informal health providers and a prevalent preference for their services among urban slum dwellers, it becomes imperative to recognize and leverage the potential contributions of informal health providers.

To harness this potential effectively, a critical step forward involves establishing transparent and well-documented processes that seamlessly integrate informal health providers into formal health systems. This collaborative synergy presents a promising avenue for addressing the healthcare challenges specific to urban slums. With this, there is a tangible opportunity to significantly enhance access to high-quality, effective, and efficient healthcare services, particularly within slums. This approach not only bridges existing gaps in healthcare accessibility but also sets the stage for a more inclusive, responsive, and resilient healthcare system, thereby fostering improved health outcomes in urban slum settings.

## Data Availability

The data and materials used for this study are available from the corresponding author on reasonable request.
